# Annotated digital image correlation displacement fields from fatigue crack growth experiments on aluminium alloys

**DOI:** 10.1038/s41597-026-07722-1

**Published:** 2026-06-26

**Authors:** David Melching, Ferdinand Dömling, Florian Paysan, Erik Schultheis, Eric Dietrich, Eric Breitbarth

**Affiliations:** Institute for Frontier Materials on Earth and in Space, German Aerospace Center (DLR), Linder Hoehe, Cologne, 51147 Germany

## Abstract

We present a curated dataset of planar displacement fields from eight fatigue crack growth experiments obtained via full-field digital image correlation (DIC). The dataset covers multiple aerospace-grade aluminium alloys, specimen geometries, material orientations, and load configurations, providing a diverse experimental basis for data-driven fracture mechanics research. Crack tip locations are consistently annotated using an iterative correction procedure applied to all measurements, and fracture mechanical descriptors like stress-intensity factors are provided as additional labels. The dataset comprises 8,794 unique experimentally observed displacement fields and a total of 70,352 supervised samples generated through standardized interpolation and augmentation. DIC data is provided as uniformly interpolated displacement grids at three standardized resolutions (28 × 28, 64 × 64, and 128 × 128 pixels), each available in three dataset sizes to support scalable use cases ranging from educational applications to high-capacity model development. Accompanying metadata and a Python interface facilitate filtering, loading, and integration into reproducible machine learning and fracture mechanics workflows.

## Background & Summary

Fatigue crack growth (FCG) experiments are fundamental in materials science and structural engineering because they inform lifetime prediction and safe design. Conventional crack length measurements, such as the potential drop technique, provide robust estimates but lack the spatial detail needed to analyse local crack growth mechanisms. Analytical solutions and numerical simulations can estimate the stress state near the crack-tip, but they rely on idealised assumptions and therefore provide only indirect insight into local crack-tip behaviour. Optical methods such as digital image correlation (DIC) bridge this gap. DIC delivers planar full-field displacement maps throughout crack propagation, supporting quantitative analysis of near-crack-tip fracture mechanics as well as global full-field analysis of boundary conditions and introduced forces. Robust crack tip localization in DIC remains challenging due to measurement noise, limited spatial resolution, and the subtle gradients that characterize the elastoplastic fields near the tip.

In parallel, machine learning (ML) and deep learning (DL) approaches have gained increasing attention in fracture mechanics. Convolutional neural networks (CNNs) have been used to predict full-field stress distributions and stress concentrations in cracked or damaged structures^1^. Graph-based and transfer-learning frameworks have been developed to emulate crack propagation and stress intensity factors in brittle fracture problems^2^. Interpretable machine learning approaches like symbolic regression have also been proposed to construct surrogate models for stress intensity factors by learning corrections to classical analytical solutions^3^. More recently, physics-enhanced deep learning models have been introduced to directly predict stress intensity factors for complex crack configurations by combining fracture-mechanics features with CNNs^4^. However, the training data underpinning these approaches are exclusively derived from numerical simulations, often based on idealized geometries, simplified material behavior, and noise-free fields. While such synthetic datasets are well suited for proof-of-concept studies, they only partially reflect the complexities of experimental measurements, including optical noise, spatial resolution limits, and deviations from ideal boundary conditions. More recently, deep-learning-based approaches have been proposed for crack path and crack tip detection in experimental settings involving DIC data^5,6^, as well as physics-informed iterative correction schemes achieving sub-pixel crack tip accuracy^7^. Although these studies clearly demonstrate the potential of data-driven and hybrid methods for crack-tip analysis in DIC, their broader comparison, reproducibility, and systematic benchmarking remain constrained by the absence of standardized, openly available, and consistently annotated experimental datasets based on full-field measurements. Existing dataset-focused contributions in the field, such as Mechanical MNIST Crack Path^8^ or large-scale fracture simulation repositories^9^, are likewise limited to synthetic data, underscoring the need for experimentally grounded benchmarks that capture the real-world challenges of DIC-based fatigue crack growth analysis.

This work addresses this need by assembling a curated and annotated dataset called *CrackMNIST*, which consists of full-field displacement data acquired during eight FCG experiments with a fully automated robotic test stand and a commercial Zeiss Aramis system^10,11^. Parts of the underlying experimental data and analysis workflows have previously been used to investigate automated fatigue testing^11^, crack growth monitoring^5,6^ and fracture mechanical evaluations^10,12–15^. However, these earlier works did not provide a unified, machine-learning-ready benchmark dataset with consistently processed displacement fields, standardized train/validation/test splits, multiple interpolation-grid resolutions, crack-tip segmentation masks, stress-intensity-factor labels, metadata, and a dedicated Python interface. The novelty of the present work is therefore the consolidation, harmonization, annotation, and public release of these experimental DIC measurements as a reusable dataset for reproducible machine-learning and fracture-mechanics benchmarking. Ground-truth crack tip positions are provided via an iterative correction procedure applied consistently across all experiments^7^. In addition to crack tip coordinates, the dataset includes fracture-mechanics-related quantities derived from the near-tip displacement fields. In particular, stress intensity factors are provided as annotations, enabling supervised learning approaches that map DIC displacement fields directly to physically meaningful fracture descriptors. To support a broad range of use cases, from rapid prototyping to high-fidelity modelling, the dataset features interpolated DIC displacement fields at various resolutions ranging from 28 × 28 to 128 × 128 pixels, all representing the same physical region of roughly 40 − 60 mm × 40 − 60 mm on the specimen. For each resolution, three dataset sizes (S, M, and L) are provided, enabling flexible scalability for algorithm development and offering lightweight subsets tailored to educational applications.

To summarize, the published per-resolution-datasets provide a total of 70,352 supervised samples derived from 8,794 unique experimentally observed displacement fields.

Key characteristics of the dataset include:

### Diversity

Eight experiments spanning AA2024, AA7475, and AA7010 alloys and covering multiple specimen geometries, thicknesses, fracture toughness orientations, and load ratios.

### Scalability

The DIC displacement fields are interpolated to regular grids at several pixel resoultions, 28 × 28, 64 × 64, and 128 × 128 pixels. Each resolution comes in three dataset sizes (S, M, L) supporting both lightweight exploration at a low entry-barrier as well as high-capacity, high-fidelity model training.

### Reuse potential

The dataset supports supervised learning tasks including crack tip localization and the direct regression of fracture-mechanics descriptors such as stress intensity factors from displacement fields, as well as studies on resolution dependence, data efficiency, uncertainty quantification.

### Education

The consistent structure, varied experimental cases, and multiple resolutions, particularly the 28 × 28 variant, make the dataset suitable for teaching modern machine-learning-based computer vision techniques using experimentally acquired fracture mechanics data.

Although *CrackMNIST* provides a comparatively large number of machine-learning samples, its experimental scope remains deliberately focused. The 70,352 augmented samples are derived from 8,794 unique DIC displacement fields acquired in eight fatigue crack growth experiments. They should therefore not be interpreted as 70,352 statistically independent experiments. The dataset currently covers three aerospace-grade aluminium alloys, two specimen geometries, and a limited set of load ratios. As a consequence, the dataset is best suited for benchmarking crack-tip localization and fracture-mechanics regression tasks within the domain of aluminium fatigue crack growth. Direct generalization to other material classes, such as steels, titanium alloys, nickel-base superalloys, or high-temperature systems, should be treated with caution because these materials may exhibit different deformation mechanisms, crack-tip plasticity, damage processes.

## Methods

**Fig. 1:**
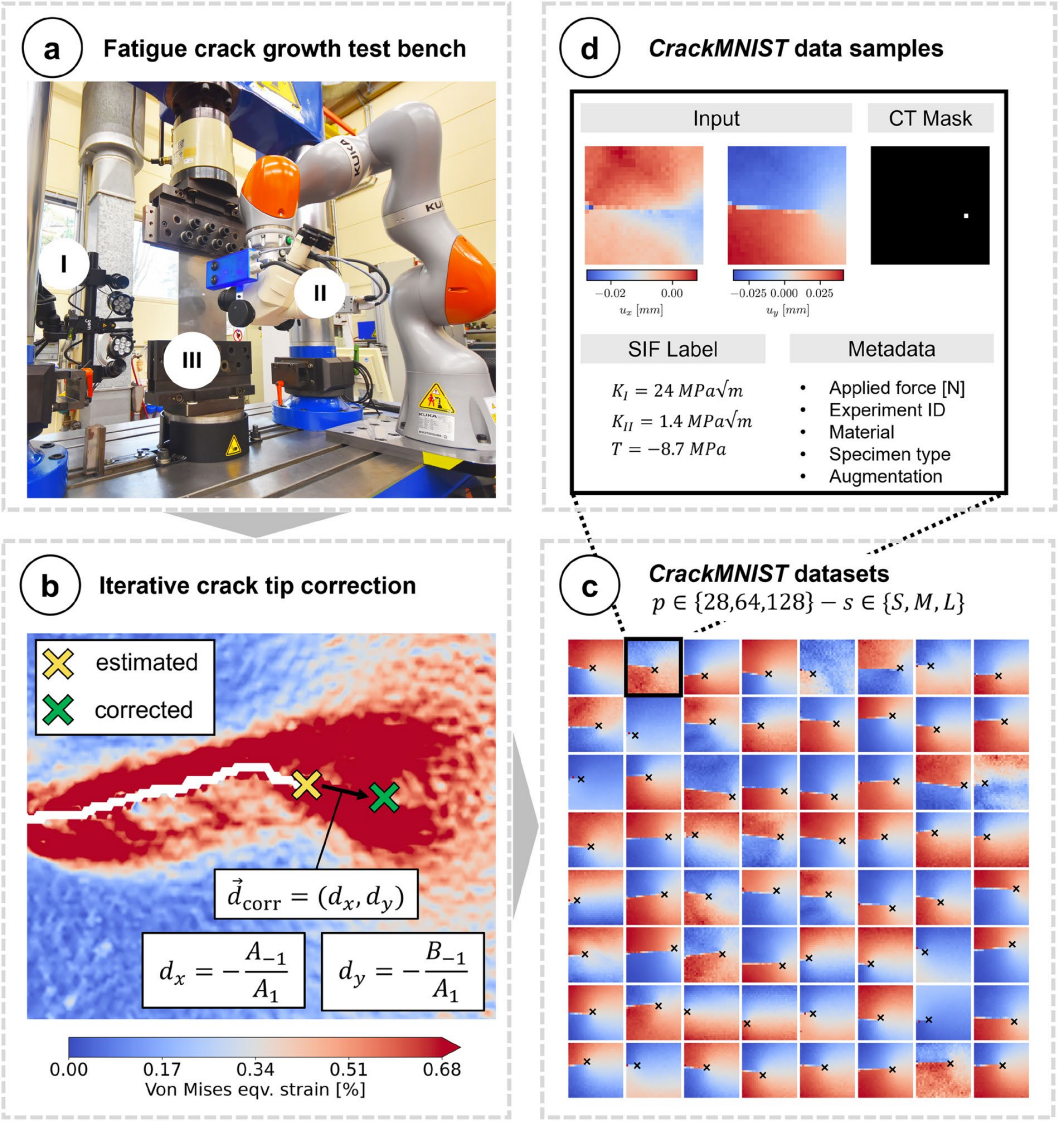
Overview of the *CrackMNIST* dataset creation workflow: **(a)** Experimental setup for fatigue crack growth testing^11^ with **I**: full-field 3D DIC by a commercial Zeiss Aramis system, **II**: high-resolution DIC option using a KUKA collaborative robot with a global shutter camera (not used in this dataset release), and **III**: material specimen (here: MT160). **(b)** Crack tip annotation process showing initial estimate (yellow cross), refined position after iterative correction (green cross), and the symbolic regression correction formulas applied to Williams-series coefficients. **(c)** Examples of processed displacement field samples at 28 × 28 pixel resolution with crack tip labels (black crosses) as provided in the *CrackMNIST* dataset. The dataset is available at pixel resolutions, *p* ∈ {28, 64, 128}, and with various sizes, *s* ∈ {*S*, *M*, *L*}. **(d)** Data samples consist of input displacement field (*u*_*x*_, *u*_*y*_) with corresponding target crack tip masks and stress intensity factors. For each sample metadata such as the applied external force and specimen and material type are provided.

The creation of *CrackMNIST* followed a structured workflow from controlled fatigue crack growth experiments to standardized machine-learning-ready datasets (Fig. 1). Full-field 3D digital image correlation (DIC) measurements were acquired on aerospace-grade aluminium alloy specimens. Crack tip positions were then annotated with a high-accuracy iterative correction procedure based on fitting the experimental full-field displacements to the theoretical Williams field leading to estimated crack tip accuracies of 0.02 mm, cf. Section “Crack Tip Annotation”. Mode-I and mode-II stress intensity factors, *K*_*I*_, *K*_*II*_, together with the T-stress, *T*, were calculated by fitting the Williams series expansion using *CrackPy*^16^. Finally, both displacement fields were randomly augmented and interpolated to multiple pixel resolutions.

### Experimental campaign

Fatigue crack growth experiments were conducted in accordance with ASTM E647-15^17^ using a servo-hydraulic uniaxial testing machine. A Zeiss Aramis 3D DIC system observed the rear side while a robotic high-resolution 2D (HR-DIC) system was used to track the front side crack-tip region for detailed investigation of the plastic zone. For the *CrackMNIST* dataset, only the global 3D DIC data are considered, as the HR-DIC data is restricted to a highly localized field of view.

The conducted experiments are summarised in Table 1, covering aluminium alloys AA2024, AA7475, and AA7010 in rolled (*r*) or forged (*f*) condition, with MT160 and CT75 specimen geometries, thicknesses between 2–12 mm, and multiple fracture orientations (LT, TL, SL45° following ASTM nomenclature for material orientation^18^) and load ratios *R*. For this test campaign, the maximum load ${F}_{\max }$ was kept constant for all *R*, while the minimum load ${F}_{\min }$ was adjusted to achieve the prescribed *R*-ratio. All tests were performed under sinusoidal, constant-amplitude loading with a frequency of 20 Hz.

**Table 1:** Experiments included in *CrackMNIST* consist of different materials, specimen types, specimen thicknesses, fracture toughness orientations, stress ratios *R*. ^*r*^Rolled, ^*f*^Forged.

Experiment	Material	Specimen Type	Thickness [mm]	Orientation	R
MT160_2024_LT_1	AA2024-T3^*r*^	MT160	2	LT	0.1
MT160_2024_LT_2	AA2024-T3^*r*^	MT160	2	LT	0.3
MT160_2024_LT_3	AA2024-T3^*r*^	MT160	2	LT	0.5
MT160_2024_TL_1	AA2024-T3^*r*^	MT160	2	TL	0.1
MT160_2024_TL_2	AA2024-T3^*r*^	MT160	2	TL	0.3
MT160_7475_LT_1	AA7475-T761^*r*^	MT160	4	LT	0.1
MT160_7475_TL_1	AA7475-T761^*r*^	MT160	4	TL	0.3
CT75_7010_SL45_1	AA7010-T7451^*f*^	CT75	12	SL45°	0.1

### Digital image correlation system

The full-field 3D DIC system (Zeiss Aramis 12M) comprised two 12 MP cameras (4 619  × 2 598 pixels) mounted with a slider distance of 98 mm and a mutual viewing angle of 25° on a stereo base. The measurement volume was configured to 200 × 150 × 21 mm^3^ using 50 mm lenses, with the camera pair positioned 525 mm from the specimen surface. Uniform illumination was provided by two 20 W, blue-light (455-475 nm) LED-spotlights. The specimen backside was prepared with a stochastic, high-contrast vanish spray pattern, suitable for DIC feature tracking. The DIC facet size used during correlation should be distinguished from the pixel resolution of the released *CrackMNIST* images. The Aramis evaluation was performed on the native camera images using a facet size of 19 × 19 camera pixels, corresponding to approximately 0.86 × 0.86 mm^2^, and a facet spacing of 16 camera pixels, corresponding to approximately 0.59 × 0.59 mm^2^. These parameters define the spatial sampling and measurement robustness of the original DIC displacement fields. The subsequently released 28 × 28, 64 × 64, and 128 × 128 *CrackMNIST* arrays are generated only after DIC evaluation by interpolation of the already-computed displacement fields. They therefore do not represent alternative DIC subset sizes and do not change the underlying DIC measurement accuracy. In accordance with established DIC practice, the subset/facet size must be selected relative to the speckle pattern, optical magnification, expected displacement gradients, and required displacement accuracy. A sufficiently large subset is required to contain several independent speckle features and to ensure robust correlation, whereas too large a subset reduces the spatial resolution of the measured displacement field.

In Table 2 user-accessible evaluation settings are reported. Proprietary implementation details of the internal image correlation algorithm are not exposed by the software and therefore not available to the authors.

**Table 2:** Summary of the user-accessible DIC hard- and software parameters used for the evaluation of the datasets.

DIC System	ZEISS GOM Aramis 12 M
Image Size	4 619 × 2 598 px^2^
Focal Length	50 mm
Measurement-Volume	200 × 150 × 21mm^3^
Stereo-Angle	25°
Stand-Off Distance	525 mm
Lighting	2 × 20 W, blue-light (455-475 nm) LED-Spotlights
DIC Software	GOM ARAMIS Professional 2020, ZEISS/GOM
Reference Image	Single image at Force <200 N (standard correlation)
Interpolant	Bi-cubic spline
Subset/Facet Size	19 px (0.86 mm)
Point Distance	16 px (0.59 mm)
Patterning Technique	Base coat of white spray varnish with black iron oxide pigment speckles
Pattern Feature Size	≈6 px (0.2 mm)

### Data acquisition

During testing, a direct current potential drop (DCPD) system monitored crack length in real time and triggered image acquisition. After each crack growth increment of Δ*a*_step_ = 0.5 mm, the DIC systems recorded data at at least three discrete loads corresponding to the minimum ${F}_{\min }$, an intermediate $({F}_{\min }+{F}_{\max })/2$, and the maximum ${F}_{\max }$ load level. This automation enabled the acquisition of spatially and temporally resolved displacement fields throughout each experiment.

### Crack tip annotation

Crack tip positions were annotated in physical coordinates (mm) on the raw DIC data using a three-stage CrackPy^16^ workflow. First, the displacements, strains and derived stresses were processed for each specimen side, with the left side mirrored to ensure a consistent coordinate system. An initial crack path, angle, and tip estimate (*x*_0_, *y*_0_, *θ*) was obtained using a line-intercept detector operating on a regular grid of probe lines with a spacing of 0.1 mm in both the *x*- and *y*-directions. To ensure a physically correct crack tip position, this rough estimate was then refined by iterative fitting of the Williams series to the measured fields, using the correction formulas discovered by symbolic regression in^7^:

1 $${d}_{x}=-\frac{{A}_{-1}}{{A}_{1}},\quad {d}_{y}=-\frac{{B}_{-1}}{{A}_{1}},\quad \varepsilon =\sqrt{{A}_{-1}^{2}+{B}_{-1}^{2}},\quad \delta =\sqrt{{d}_{x}^{2}+{d}_{y}^{2}}.$$

The iteration was terminated once the correction magnitude *δ* fell below 5 ⋅ 10^−3^ mm. Finally, the correction with the smallest error, *ε*, was selected. Analysis of the convergence behavior reported in^7^ indicates geometrically shrinking corrections with a contraction factor *q* ≈ 0.6 − 0.8. Under this assumption, the residual numerical uncertainty due to early stopping is bounded by *δ*/(1 − *q*), corresponding to an expected termination error of approximately 0.02 mm for the chosen tolerance. This numerical contribution is small compared to the intrinsic model and measurement uncertainty of the Williams-fit-based correction reported in^7^. The estimated crack-tip accuracy of approximately 0.02 mm reported above refers to the numerical contribution associated with the stopping criterion of the iterative correction procedure. It should not be interpreted as the complete experimental uncertainty of the crack-tip labels. In addition, the total uncertainty depends many factors such as DIC displacement noise, speckle pattern quality, the chosen fitting region, near-tip plasticity or closure effects. All annotations were performed on the native DIC facet grid and subsequently mapped to the standardized interpolated image grids (28 × 28, 64 × 64, 128 × 128) by applying the same affine transformation used for displacement interpolation. The labels are provided as binary segmentation masks with the tip pixel marked positive.

### SIF annotation

Stress intensity factors (SIFs) *K*_I_, *K*_II_, and the non-singular *T*-stress were annotated for each data point by fitting the Williams series to the measured DIC displacement fields using the CrackPy fracture analysis framework^16^. Starting from the refined crack-tip position and crack angle obtained during crack tip annotation, the fracture analysis workflow performed an iterative least-squares optimization of the Williams expansion over a prescribed set of terms and radial fitting bounds. For each data point, the pipeline evaluated the analytical displacement fields for the selected Williams modes and minimized the residuals to determine the coefficients *A*_*n*_ and *B*_*n*_. The mode-I and mode-II SIFs and T-stress were then computed directly from the coefficients as

2 $${K}_{{\rm{I}}}=\sqrt{2\pi }\,{A}_{1},\qquad {K}_{{\rm{II}}}=-\sqrt{2\pi }\,{B}_{1},\qquad T=4{A}_{2}.$$

The SIF annotations are derived quantities obtained by fitting Williams-series displacement fields to experimental DIC data. Their uncertainty is therefore affected by the displacement-field noise, the annotated crack-tip position and crack angle, the selected Williams terms, radial fitting bounds, and the validity of the underlying linear-elastic crack-tip-field approximation for the considered region. The provided *K*_*I*_, *K*_*II*_, and *T* values should therefore be understood as consistent experimental estimates generated by a fixed analysis pipeline rather than as noise-free ground-truth quantities. A full uncertainty propagation study, for example by repeated image acquisition, perturbation of the displacement fields, or Monte-Carlo variation of crack-tip locations and fitting parameters, is outside the scope of this dataset descriptor but represents an important direction for future work.

### Metadata annotation

Each image and its corresponding crack tip mask or SIF target are accompanied by a set of metadata that extends the static, experiment-level descriptors (Table 1) with per-step information, such as applied force. This metadata is intended to enable custom training and evaluation strategies as well as physically meaningful data selection. An overview of metadata fields available for query is provided in Table 3.

**Table 3:** Overview of the provided metadata fields in *CrackMNIST*. Each Descriptor is accessible via the data loader (see *Usage Notes*).

Descriptor	Type	Values	Explanation
Experiment	Categorical	see Table 1	Unique identifier of the experiment
Material	Categorical	see Table 1	Alloy designation of the tested material
Specimen Type	Categorical	see Table 1	Geometrical specimen configuration^18^
Thickness	Categorical	see Table 1	Nominal specimen thickness
Orientation	Categorical	see Table 1	Crack plane/ Mode I loading direction relative to material rolling directions
R	Categorical	see Table 1	Nominal load ratio ($R={F}_{\min }/{F}_{\max }$) of the fatigue cycle
Side	Categorical	left, right	Imaged side relative to *pre-crack* notch geometry; distinguishes in left and right cracks for symmetric specimens (MT160).
Force	Continuous	[200, 30000] *N*	Applied uniaxial load during image acquisition.

**Fig. 2:**
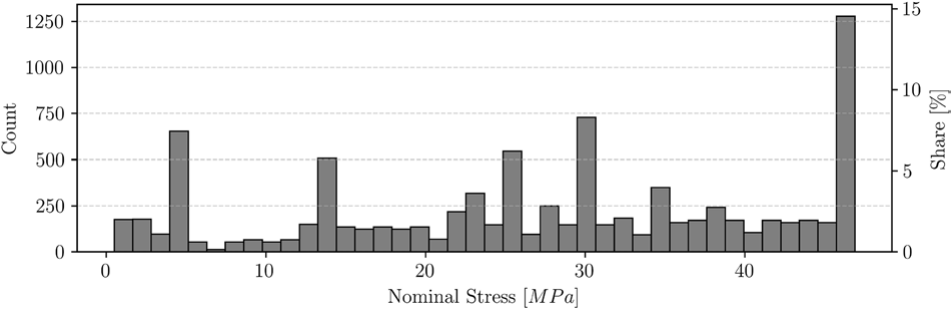
Distribution of samples across applied normalized loads. Counts are based on non-augmented data.

Table 4 summarizes the distribution of the 8,794 unique experimentally observed displacement fields across the available metadata descriptors underlying the *CrackMNIST* dataset. Figure 2 shows the distribution of the applied nominal stresses, calculated as applied external force divided by the product of specimen thickness and width, using 40 bins. Of all nominal stresses, 43% fall into bins corresponding to ${\sigma }_{\min }$, $({\sigma }_{\min }+{\sigma }_{\max })/2$ and ${\sigma }_{\max }$ in equal proportions. The majority of 57% is accounted for by further intermediate loads between ${\sigma }_{\min }$ and ${\sigma }_{\max }$.

**Table 4:** Distribution of samples across categorical metadata descriptors. footnoteCounts are based on non-augmented data.

Descriptor	Value	Count	Share [%]
experiment	MT160_2024_TL_2	1486	16.90
	MT160_7475_LT_1	1454	16.53
	MT160_2024_LT_2	1276	14.51
	MT160_2024_LT_1	1276	14.51
	MT160_2024_LT_3	1232	14.01
	MT160_2024_TL_1	743	8.45
	MT160_7475_TL_1	716	8.14
	CT75_7010_SL45_1	611	6.95
material	AA2024 (rolled)	6013	68.38
	AA7475 (rolled)	2170	24.68
	AA7010 (forged)	611	6.95
specimen_type	MT160	8183	93.05
	CT75	611	6.95
thickness_mm	2	6013	68.38
	4	2170	24.68
	12	611	6.95
orientation	LT	5238	59.56
	TL	2945	33.49
	SL45°	611	6.95
R	0.1	4084	46.44
	0.3	3478	39.55
	0.5	1232	14.01
side	right	4721	53.68
	left	4073	46.32

### Resolutions and interpolation

**Fig. 3:**
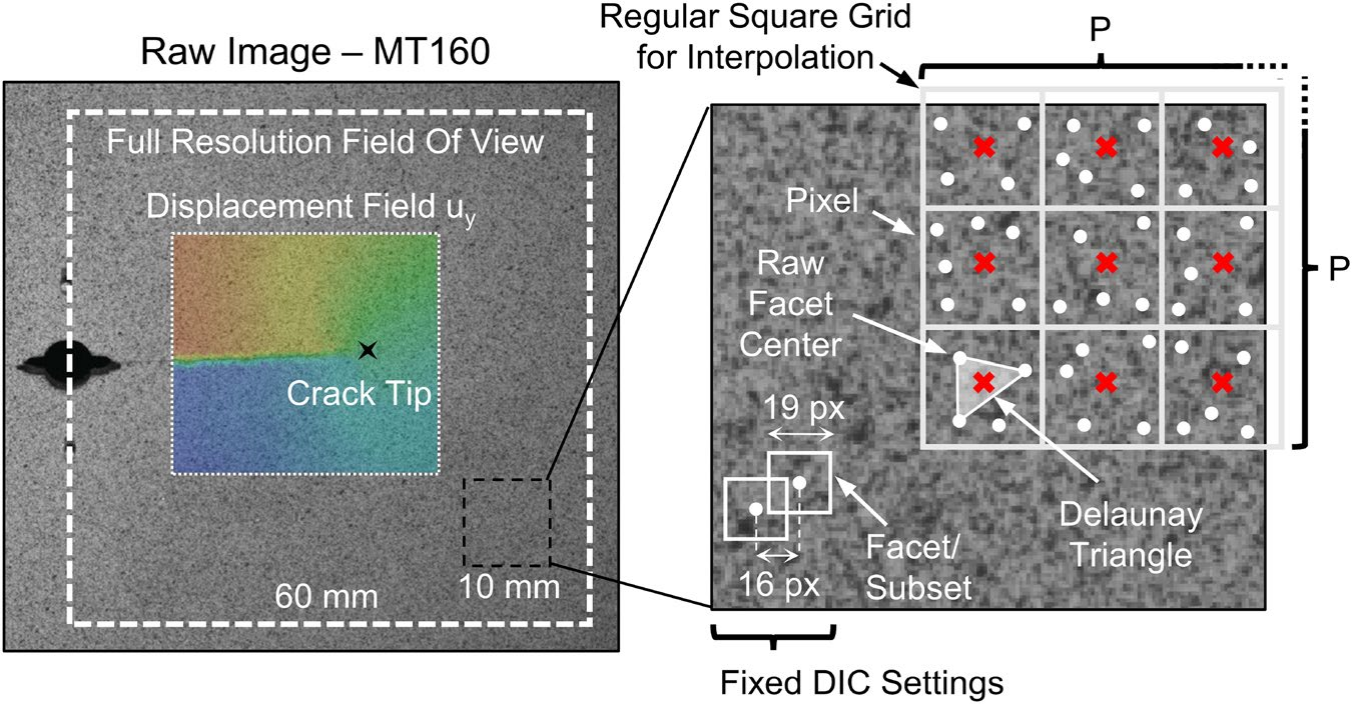
Schematic overview of the procedure for processing raw DIC data into interpolated data in *CrackMNIST*. Left: Raw image and full-resolution field of view (60 × 60, mm^2^) serving as the basis for interpolation. Right: Magnified view of the specimen speckle pattern with indicated DIC subset/facet size, evaluated facet centers, Delaunay triangulation and regular square evaluation grid used for subsequent linear interpolation.

The released displacement fields are experimental DIC measurements and therefore contain measurement uncertainty. Relevant sources include camera image noise, calibration and stereo-reconstruction uncertainty, speckle-pattern quality, lighting variations, facet size and spacing, local decorrelation close to the crack flanks or specimen boundaries, and interpolation from the native DIC facet grid to the standardized *CrackMNIST* grids. Interpolation changes the sampling grid used by the machine-learning model, but it does not reduce the original DIC measurement uncertainty or create additional independent measurements. All annotations and displacement fields were generated on the raw DIC facet grid obtained from the Zeiss Aramis system. For machine learning applications and standardization across experiments, the raw fields were interpolated onto equidistant square grids of size *P* × *P*, with *P* ∈ {28, 64, 128}. The resulting grids represent a physical field of view of approximately 40 × 40 mm^2^ and 60 × 60 mm^2^ for the CT- and MT-specimen, respectively. Interpolation was performed using bilinear mapping in the specimen’s physical coordinate frame, ensuring that both displacement components (*u*_*x*_, *u*_*y*_) and crack tip positions were transformed consistently. The lower resolutions provide a lightweight *MNIST*-style dataset suitable for educational purposes and rapid prototyping, while higher resolutions preserve fine-scale displacement gradients essential for high-fidelity crack tip detection. Figure 3 illustrates the procedure in schematic form.

**Fig. 4:**
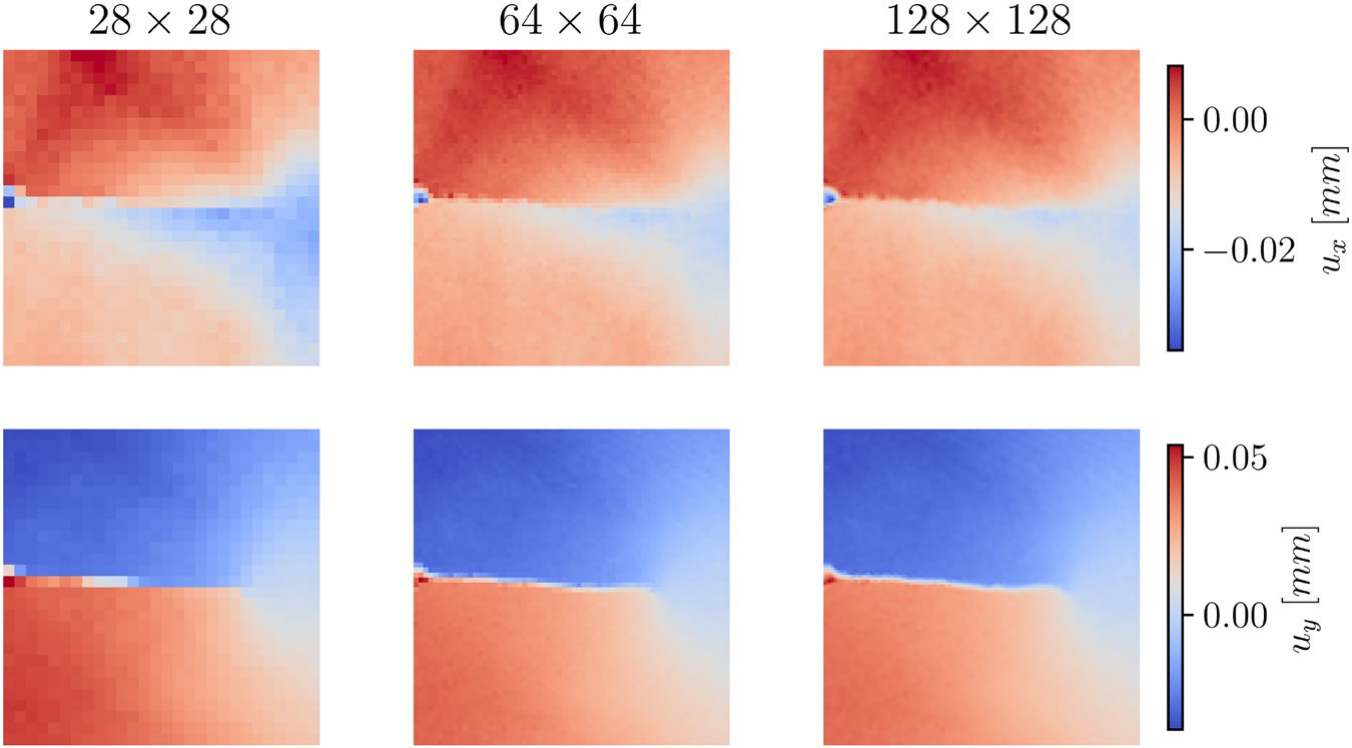
*CrackMNIST* DIC data sample interpolated to different resolutions. Top: *x*-displacement in *mm*. Bottom: *y*-displacement in *mm*. Columns show the same sample at various resolutions.

The term resolution refers exclusively to the size of the standardized machine-learning input grid. It should not be confused with the DIC subset or facet size used during correlation. Interpolation to a denser grid increases the number of pixels available to a learning algorithm, but it does not create additional independent DIC measurements beyond those provided by the original facet grid. The three resolutions are intended for different machine-learning use cases. The 28 × 28 datasets provide a compact representation for teaching, rapid prototyping, and low-resource demonstrations. The 64 × 64 datasets provide a compromise between computational cost and spatial detail and are recommended as a practical default for many benchmark studies. The 128 × 128 datasets provide a denser interpolated representation for high-capacity models and for studying the influence of input-grid density. Because all three variants originate from the same native DIC facet grid, they should not be interpreted as a DIC subset-size optimization. A true subset-size sensitivity study would require re-evaluating the original stereo image data with different facet sizes and comparing the resulting displacement uncertainty, spatial resolution, crack-tip annotations, and fracture-mechanics quantities. Such an analysis is beyond the scope of the present dataset release but represents an important direction for future work. Figure 4 shows a DIC sample datapoint at the different resolutions provided by *CrackMNIST*.

### Processing and augmentation

**Fig. 5:**
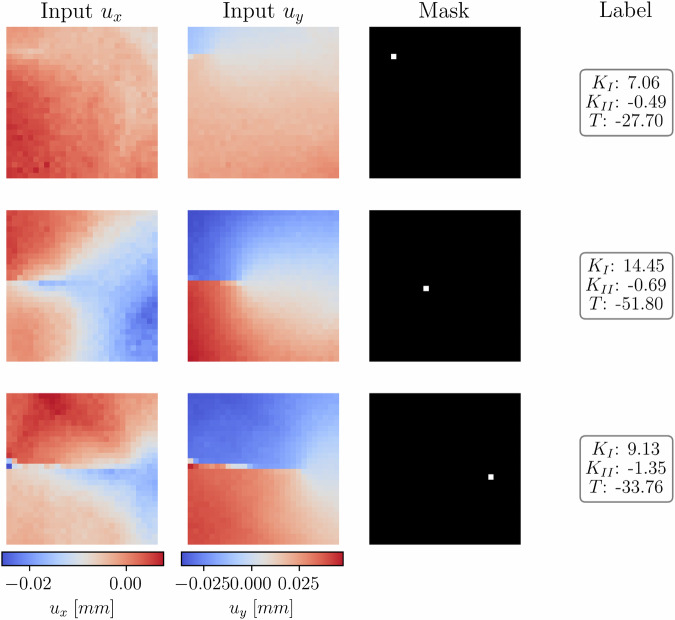
Three random *CrackMNIST* samples at a resolution of 28 × 28 pixels. Each input consists of two channels representing the in-plane displacement components, *u*_*x*_ and *u*_*y*_, while the corresponding binary segmentation masks indicate the crack tip location, shown as white pixels. Additional annotations of the stress-intensity factors, *K*_*I*_, *K*_*II*_, both in ${\rm{MPa}}\sqrt{m}$, and the *T*-stress in MPa are given.

In general, all experiments with a middle tension (MT) specimen contain a left- and right-hand side. For consistency, the left-hand side is rotated to the right such that the crack always grows in the same direction. For each original DIC data point, 8 augmented variants are generated by applying random shifts (*x* up to 10 mm, *y* ± 10 mm), rotations (±10°), and a 50% chance of vertical flips. The augmented crack tip position is transformed accordingly, and samples producing NaNs are discarded and re-drawn. Augmentations are first validated at 256 px, then applied identically to produce aligned versions at 28, 64, 128 px. This ensures all resolutions contain *exactly the same physical samples*, differing only in interpolation grid spacing. Labels are binary masks with value 255 (white/foreground) at the crack tip and 0 (black/background) elsewhere. For higher resolutions, a single crack tip pixel occupies a negligible fraction of the image, making it difficult to detect. To mitigate this, the crack tip label is dilated by one pixel in all directions, creating a small cluster of labelled pixels around the tip to improve visibility and learning stability. Therefore, to keep the crack-tip-to-background ratio relatively constant the crack tip masks are enlarged to 3 × 3 and 5 × 5 for the resolutions 64 × 64 and 128 × 128, respectively. Inputs contain two channels: The planar displacements in *x*-direction, *u*_*x*_, and in *y*-direction, *u*_*y*_. Figure 5 displays the displacement fields and crack tip segmentation masks of *CrackMNIST* data samples with various crack lengths.

### Data splitting

To prevent data leakage, the train/validation/test split was performed at the level of experiment sides before augmentation. An experiment side denotes all DIC fields belonging to one crack side of one fatigue crack growth experiment, for example MT160_2024_LT_3_right. All original DIC displacement fields from such an experiment-side group are assigned to exactly one split. The augmented variants generated from these fields inherit the same split assignment. Consequently, no shifted, rotated, or flipped version of a training sample appears in the validation or test set. The split assignment is fixed across all three *CrackMNIST* resolutions. Thus, the same physical DIC measurement belongs to the same split for the 28 × 28, 64 × 64, and 128 × 128 datasets. This allows direct comparison of input resolutions while avoiding cross-resolution inconsistencies in the benchmark splits. The splits were intentionally defined by experiment-side groups rather than by random sample-level splitting. This design provides a more conservative benchmark, because validation and test samples originate from held-out experiment sides instead of near-duplicate augmented variants of training samples. Table 5 summarizes the dataset splits for each size (S, M, L), listing the corresponding experiments used for training, validation, and test sets.

**Table 5:** Experimental DIC data used by size and split (train, validation, test).

Size	Train	Validation	Test
S	MT160_2024_LT_1_right, MT160_2024_LT_3_right	MT160_2024_TL_2_left	MT160_2024_TL_1_right
M	MT160_2024_LT_3_right, MT160_2024_LT_3_left, MT160_2024_TL_2_right, MT160_7475_LT_1_right	MT160_2024_TL_2_left, MT160_7475_LT_1_left	MT160_2024_TL_1_right, MT160_7475_TL_1_left
L	MT160_2024_LT_1_right, MT160_2024_LT_1_left, MT160_2024_LT_2_right, MT160_2024_LT_2_left, MT160_2024_LT_3_right, MT160_2024_LT_3_left, MT160_2024_TL_2_right, MT160_7475_LT_1_right	MT160_2024_TL_2_left, MT160_7475_LT_1_left	MT160_2024_TL_1_right, CT75_7010_SL45_1_right, MT160_7475_TL_1_left

### Dataset sizes

To support scalable benchmarking and usability without large computational resources (e.g. educational demonstration and teaching), the augmented dataset comprising 70,352 unique samples is released at each resolution in three dataset sizes (S, M, L). Fixed splits are used and are identical across resolutions. After augmentation, the datasets comprise a total of 70,352 samples. The sample counts for each dataset size and data split are summarized in Table 6.

**Table 6:** Available Benchmark datasets and their sizes after the application of the augmentation strategy.

Size	Train	Validation	Test
S	10,048	5,944	5,944
M	21,640	11,736	11,672
L	42,056	11,736	16,560

## Data Record

### Repository and identifiers

The dataset is archived on Zenodo (Version 2.0.0; published Feb 4, 2026) under the 10.5281/zenodo.18454958 with a CC BY 4.0 license^19^. The record provides file-level MD5 checksums and a total hosted size of 21.1 GB.

### File inventory and structure

Data are distributed as standalone HDF5 files named by pixel resolution and dataset size: crackmnist_{28,64,128}_{S, M, L}.h5. The Zenodo record hosts nine files for 28, 64, and 128 px (each with S, M, L). Each HDF5 file is self-contained; there is no nested directory structure besides the split organization described below.

### Contents and format

Within each HDF5, samples are organized into three splits—train, val, and test. For every sample, the inputs are planar displacement fields *u*_*x*_, *u*_*y*_ (in mm) derived from digital image correlation, and the target is - depending on the chosen learning task - either a binary segmentation mask indicating the crack tip location or a vector containing (*K*_*I*_, *K*_*II*_, *T*). The files are provided for three pixel resolutions (28 × 28, 64 × 64, 128 × 128). The JSON file experiments_metadata.json contains the experiment metadata, i.e. material and specimen types and thicknesses as well as fracture orientations and load ratios.

## Technical Validation

The technical validation of the dataset focused on verifying the integrity, consistency, and physical plausibility of the released data. The validation checks were applied to the HDF5 files, metadata, displacement fields, crack-tip masks, stress-intensity-factor labels, augmentation records, and fixed train/validation/test splits. The machine learing functionality of the dataset is demonstrated through extended baseline scripts in the corresponding repository^20^.

### File integrity and HDF5 structure

All released HDF5 files were checked for readability and internal consistency. For each dataset size and pixel resolution, the expected train, validation, and test groups were verified to be present. The file-level checksums provided in the data repository allow users to verify download integrity.

### Metadata consistency

The experiment identifiers stored in the HDF5 files were checked against the accompanying metadata file. The material, specimen type, specimen thickness, fracture orientation, load ratio, specimen side, and force entries were verified to be consistent with the experimental campaign. The distributions of categorical metadata descriptors and applied nominal stresses were inspected to identify missing, duplicated, or inconsistent entries. These checks confirmed that each sample can be traced back to its corresponding experiment and specimen-side configuration.

### Split integrity and leakage prevention

The splits were checked at the level of experiment sides. All original DIC displacement fields from one experiment side are assigned to exactly one split, and all augmented variants inherit the same split assignment. Thus, no shifted, rotated, or flipped version of a training sample appears in more than one split preventing data leakage.

### Interpolation and augmentation consistency

The interpolation workflow was checked by verifying that each physical DIC measurement is represented consistently at all three released resolutions. The transformed crack-tip coordinates were checked against the interpolated displacement fields and the corresponding crack-tip masks. Augmentation records were checked for valid translations, rotations, and flips. Samples that produced invalid values during interpolation were discarded and regenerated during dataset creation.

### Crack-tip-label validation

**Fig. 6:**
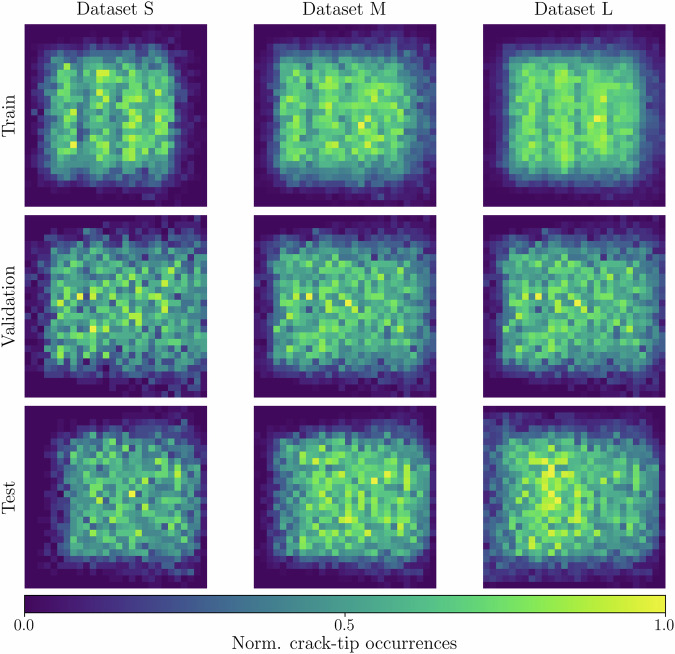
Spatial occurrence of crack-tip labels for the 28 × 28 CrackMNIST datasets. Columns correspond to dataset sizes S, M, and L; rows correspond to the training, validation, and test splits. Each panel shows the independently *per-panel* min-max normalized accumulated number of crack-tip foreground pixels at each image location. The maps were used as a label-consistency check to verify that the crack-tip annotations are located within the expected field of view and to inspect how the experiment-side-based split strategy affects the spatial distribution of crack-tip labels.

The crack-tip masks were checked for valid foreground labels and for consistency with the transformed physical crack-tip coordinates. For the different released resolutions, the expected mask sizes were verified: a single-pixel crack-tip label for 28 × 28 inputs, a 3 × 3 foreground region for 64 × 64 inputs, and a 5 × 5 foreground region for 128 × 128 inputs. This check ensured that the crack-tip labels are defined consistently across resolutions while accounting for class imbalance issues which would increase with higher resolutions. For the 28 × 28 data, the spatial occurrence of crack-tip labels was additionally accumulated for each dataset size and split. The resulting occurrence maps provide a compact check of the spatial coverage of the crack-tip annotations and reveal how the experiment-side-based splits distribute crack-tip locations across training, validation, and test data (Fig. 6).

### SIF-label plausibility

**Fig. 7:**
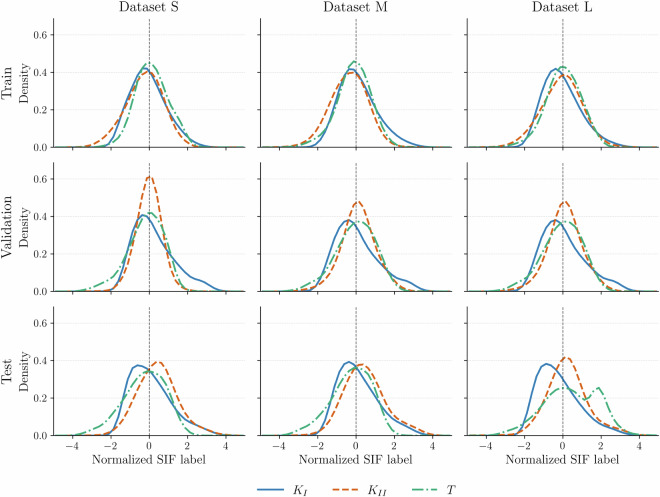
Normalized SIF label distributions for the 28 × 28 CrackMNIST datasets. Columns correspond to dataset sizes S, M, and L; rows correspond to the training, validation, and test splits. Each panel shows three density curves for the normalized stress intensity factor labels *K*_*I*_, *K*_*II*_, and *T*. Labels are z-normalized with a single mean and standard deviation computed over all splits and dataset sizes. The curves were used as a label-distribution check to compare the relative location, spread, skew, and split-dependent differences of the SIF regression targets. Because the split is performed at the experiment-side level and the datasets S, M, and L contain different sample selections, the normalized label distributions are not expected to be identical across datasets or splits.

The stress-intensity-factor labels *K*_*I*_, *K*_*II*_, and the *T*-stress were checked for finite values and physically plausible ranges under the nominal mode-I fatigue-crack-growth loading conditions of the experiments. The labels were also checked for consistency with the fixed analysis pipeline used to fit the Williams expansion to the experimental displacement fields. Since these quantities are derived from experimental DIC data, they should be interpreted as consistent experimental estimates rather than noise-free ground-truth values. Figure 7 displays the normalized SIF labels *K*_*I*_, *K*_*II*_, and *T*. Labels are z-normalized with a single shared normalization computed over all splits and dataset sizes. The resulting density curves provide a compact check of the label distributions revealing differences in location, spread, and imbalance across training, validation, and test data.

### Machine-readability checks

As a final technical check, the released data were loaded through the CrackMNIST Python interface for all dataset sizes, resolutions, splits, and target types. Inputs, masks, SIF labels, metadata, force values, and augmentation records were retrieved successfully. Simple supervised-learning runs were performed as machine-readability and label-consistency sanity checks, verifying that the released arrays and targets can be processed by standard PyTorch workflows. Detailed benchmark analyses are outside the scope of this Data Descriptor and are reported separately.

## Usage Notes

*CrackMNIST* can be accessed either (i) via the accompanying Python package crackmnist^20^ which downloads the requested dataset file fully automatically on demand, or (ii) by manually downloading the released HDF5 files and placing them into a local dataset root directory.

By default, the package uses a user cache directory (typically ~/.crackmnist on Linux) as the dataset root. For each configuration, the package expects the corresponding HDF5 file crackmnist_{pixels}_{size}.h5, where pixels ∈ {28, 64, 128} and size ∈ {*”S”*, *”M”*, *”L”*}, and the experiment metadata file experiments_metadata.json to be present in the dataset root.

### File format and dataset keys

The released data are stored in HDF5. Each file contains split-specific arrays for inputs and targets:

- {split}_images: displacement fields (two channels).
- {split}_masks: binary crack-tip masks (segmentation task).
- {split}_SIFs: (*K*_*I*_, *K*_*II*_, *T*) targets (regression task).
- {split}_exp_ids: experiment identifiers for each sample.
- experiments: experiment name strings indexed by exp_ids.
- {split}_forces: scalar load/force value per sample (see accompanying metadata for definition).
- {split}_augs: augmentation parameters applied to each sample.

The companion JSON file experiments_metadata.json stores per-experiment metadata and can be queried through the dataset helper get_metadata(idx). The functions get_forces(idx) and get_augmentations(idx) provide convenient access to per-sample forces and augmentation parameters.

### Augmentations and transforms

For each sample, the augmentation record contains shift, rotation, and a vertical flip flag. Specifically, get_augmentations(idx) returns a dictionary with shift=(shift_x, shift_y) in millimetres, rotation in degrees, and vertical_flip as a boolean indicator. These values can be used to reproduce or stratify analyses with respect to the applied geometric perturbations. The package optionally supports input/target transforms via transform and target_transform. The provided InputNormalization transform performs per-sample, per-channel standardization (i.e., normalization parameters are computed from each sample unless fixed means/stds are provided).

Minimal usage examples can be found on the GitHub repository^20^.

## Data Availability

All data described in this manuscript are openly available in the Zenodo repository under a CC BY 4.0 license at 10.5281/zenodo.18454958.

## Code availability

*CrackMNIST* is published as an open-source Python package (https://pypi.org/project/crackmnist/). The codebase is available on GitHub (https://github.com/dlr-wf/crackmnist). The data annotation algorithm is published and implemented in CrackPy (https://github.com/dlr-wf/crackpy).
